# Epidemiological insights into bovine helminthiasis in Upper Egypt: Prevalence, risk factors, and pathological findings

**DOI:** 10.17221/112/2024-VETMED

**Published:** 2025-10-31

**Authors:** Abeer Ali Khedr, Sherief Mohamed Abdel-Raheem, Mohsen Mohamed Farghaly, Saad Ibrahim Alsultan, Mohamad Abdulmohsen, Sayed Fathi El-Hawari, Wafaa Gamal El-Dein Mahmoud

**Affiliations:** ^1^Department of Parasitology, Faculty of Veterinary Medicine, New Valley University, New Valley, Egypt; ^2^Parasitology Laboratory, Department of Bioscience, Durham University, Durham, United Kingdom; ^3^Department of Public Health, College of Veterinary Medicine, King Faisal University, Al-Hofuf, Al-Ahsa, Saudi Arabia; ^4^Department of Animal Production, Faculty of Agriculture, Assiut University, Assiut, Egypt; ^5^Department of Clinical Science, College of Veterinary Medicine, King Faisal University, Al-Hofuf, Al-Ahsa, Saudi Arabia

**Keywords:** cattle, deworming efficacy, helminthic infestations, microscopic analysis, pathological examination

## Abstract

This study examines the prevalence of helminthic infestations, risk factors, and odds ratios in 1 300 cattle, revealing significant patterns in parasite distribution and influencing factors. Overall, 60.3% of cattle were infested with one or more types of parasites, with *Moniezia* spp. being the most prevalent (46.9%), followed by *Fasciola* spp. (36.9%), *Paramphistomum* spp. (26.8%), and *Avitellina* spp. (10.8%). Among the seasons, winter exhibited the highest infestation rate (66.1%), and calves under one year of age were more commonly infested (64.2%) than older cattle (over three years: 51.1%, OR = 0.584 1). Distinct seasonal and age-related patterns were observed for specific parasites. *Fasciola* spp. was most prevalent in winter (45.09%) and among the youngest cattle (47.87%), while *Paramphistomum* spp. and *Moniezia* spp. showed less variation across seasons. *Avitellina* spp. had the lowest infestation rates in the spring, and cattle older than three years were affected. Co-infections were common, notably between Fasciola and other parasites, with the highest co-infestation rate observed between *Avitellina* spp. and *Moniezia* spp. Analysis of deworming efficacy indicated higher treatment success for *Fasciola* spp. and *Paramphistomum* spp., lower odds of response for *Avitellina* spp., and no significant treatment effect for *Moniezia* spp. Microscopic and pathological examinations were also conducted. These results demonstrate the necessity of thorough deworming procedures along with targeted parasite control to reduce significant health hazards in cattle populations.

The bovine livestock sector in Egypt is a cornerstone of the agricultural economy, supplying essential nutrients and proteins vital to the population’s diet (Abdel Monem and Radojevic 2020). Cattle contribute significantly to global agriculture by providing meat, milk, and various by-products. In Egypt, cattle and buffaloes alone contribute approximately 23 percent to the total agricultural value, amounting to 73.5 billion EGP (FAO 2018). However, this sector faces considerable challenges, including limited natural pastures, low productivity, and the urgent need to improve health and reproductive management (Uddin and Kebreab 2020). The population of Egypt is expected to increase in the coming decades, necessitating enhanced livestock productivity to meet this growing need (Balehegn et al. 2020).

Parasitic infestations pose a significant threat to livestock health and productivity, with helminth parasites being particularly prevalent among ruminant animals worldwide (Hassan and Mohamed 2021). These parasites have a negative impact, especially in developing nations (Vercruysse et al. 2018), and they contribute to massive economic losses through feed utilisation, weight loss, increased mortality, and reduced production of milk and meat (Charlier et al. 2020). In Egypt, cattle are frequently affected by various helminth species, including trematodes such as *Fasciola* spp. (liver flukes) that inhabit the bile ducts and cause fasciolosis, leading to liver damage and production loss, and *Paramphistomum* spp. (rumen flukes) that inhabit the rumen and reticulum, often contributing to gastroenteritis in young ruminants. Additionally, cestodes (such as *Moniezia* spp*.* and *Avitellina* spp.) present in the small intestine cause irritation, nutrient malabsorption, and, in severe cases, intestinal obstruction (Hamid et al. 2023), which further complicates livestock management efforts (Ola-Fadunsin et al. 2020).

The New Valley Governorate, characterised by its arid to semi-arid landscapes, presents a unique environment that is conducive to helminth infestations. The region’s climate, ecological conditions, and husbandry practices create a complex scenario that significantly influences the incidence of parasitic worm infestations (Abdelhady et al. 2014; Beveridge 2020).

Desert pastures are often infested with infective-stage worm larvae or eggs (Dey et al. 2020), which contributes to their higher prevalence. Slaughterhouses are key locations for diagnosing and screening parasitic diseases within this region, providing valuable information on parasitic species and infestation levels (Sotohy et al. 2019).

In recent decades, there has been a growing interest in developing efficient control measures for parasitic infestations in ruminants worldwide (Khedri et al. 2021). The same applies in Egypt, where diverse climatic and managerial systems demand site-specific approaches for controlling helminths (Mahmoud et al. 2020; Felefel et al. 2023). The epidemiological importance of this issue, i.e., research on cattle helminth infestation in the New Valley Governorate, has been limited to date. An integrated application of all data required for effective control measures and for improving animal health and productivity in this region is urgently needed.

Therefore, this study aims to address this knowledge gap by investigating the prevalence and detailed characterisation of helminth infestations in cattle in the New Valley Governorate. By integrating meat inspection with advanced diagnostic techniques such as electron microscopy and histopathological examination, this research provides a thorough understanding of the epidemiology of these parasitic infestations. The findings will contribute to the development of targeted control measures, ultimately enhancing the health and productivity of cattle in this region.

## MATERIAL AND METHODS

### Ethical approval

This study received ethical approval from the Faculty of Veterinary Medicine, Assiut University, Egypt, in accordance with relevant Egyptian regulations governing research and publication (Approval No. 04-2024-300545).

### Study area

**Figure 1:**
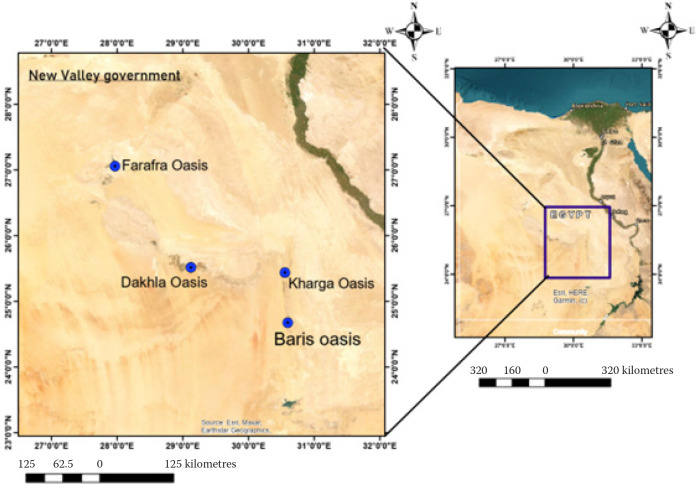
Map of the study area showing the geographical distribution of livestock

The New Valley Governorate is located in the southwestern part of Egypt at approximately 24°22'45.70''N latitude and 27°9'44.91''E longitude (Figure 1). It is the largest governorate in Egypt (440 098 km^2^) but has the lowest population density (2 persons per km^2^) and a poverty rate of 52.6%. It includes four cities: El Kharga, El Dakhla, Baris, and El Farafra Oasis. The geographical distribution of livestock components consists of 116 645 heads of cows. The management of livestock in the New Valley Governorate typically revolves around traditional methods suitable to small-scale farming (Mohammady et al. 2018).

### Cattle management practices and environmental risk factors

In the New Valley Governorate, cattle are typically raised on small-scale farms as part of mixed crop-livestock systems. Livestock is housed in small enclosures adjacent to family homes, and cared for largely by household members. Animals are mainly fed on green fodder, such as alfalfa during winter and green corn fodder (darawa), rice straw, and crop residues in summer, alongside purchased concentrates. Water is sourced primarily from groundwater wells, with irrigation provided through soaking, sprinkling, or drip systems, which also serve the needs of livestock. Animal waste is commonly disposed of near or within farm boundaries, often being used as organic fertiliser, which may contribute to environmental contamination and transmission of parasites. These conditions, including the presence of open water sources and accumulation of faecal matter, can favour the breeding of intermediate hosts such as snails and insects, thereby facilitating the life cycles of helminth parasites (Mohammady et al. 2018).

### Animals and sample collection

Between May 2023 and April 2024, a total of 5 491 cattle were slaughtered. Only cattle originating from the governorate underwent examination for helminth parasites. The study cohort included 788 males and 512 females, totalling 1 300 animals across various age groups, encompassing calves (>1 year), young cattle (1–3 years), and adults (<3 years). A total of 784 cases of helminth infestation were identified and confirmed through necropsy, gastrointestinal tract examinations and meat inspections. Epidemiological data were systematically collected on sex, age, and seasonal distribution (defined as Summer, Autumn, Winter, and Spring) (Kotsias et al. 2021), as well as the study site. Additionally, records of anthelmintic treatment within the three months to slaughter were reviewed for potential impacts on helminth infestations. in consultation with local veterinarians, nitroxinil, fenbendazole, levamisole, and oxyclozanide were identified as the most commonly used drugs. Nitroxinil**,** typically administered subcutaneously at a dose of 10 mg/kg body weight, is primarily used for the treatment of liver fluke infestation (*Fasciola hepatica*). Fenbendazole, given orally at 5–10 mg/kg, is a broad-spectrum anthelmintic effective against gastrointestinal nematodes and certain cestodes. Levamisole, commonly administered orally at 7.5 mg/kg, is used to control gastrointestinal nematodes and lungworms in cattle. Oxyclozanide, administered orally at 10–15 mg/kg, is effective against adult liver flukes and rumen flukes (*Paramphistomum* spp.). Additionally, morphological alterations and tissue pathology in recovered helminths were evaluated using scanning electron microscopy and histopathological analysis.

### Examination of the slaughtered cattle and worm collection

The post-mortem examination process started with performing a sharp visual inspection of the outer carcass surface for any visible abnormalities. Parasites detected during these inspections were carefully collected using appropriate tools to ensure thorough and meticulous examination and collection of parasites during inspections (Cooper 2021). Notably, adult helminths were only observed in the liver, rumen, and small intestines (duodenum and jejunum). A licensed veterinarian oversaw all activities at the slaughterhouse to ensure accurate observations. Following the examinations, samples were meticulously gathered, securely stored, and transported to the Laboratory of Parasitology at the Faculty of Veterinary Medicine, New Valley University, Egypt, for further detailed analysis. Before analysis, the collected specimens underwent a saline rinse to remove debris, facilitating a meticulous visual examination (Castro 1996).

### Scanning electron microscopic examination of adult helminths

Helminths isolated from the cattle that were previously treated with anthelmintic drugs were analysed for surface topography alterations, and trematodes and cestodes were detected in this study. Samples were fixed in 5% glutaraldehyde for 24–72 h, then transported to the Electron Microscopy Unit at Assiut University. They were washed using sodium cacodylate buffer followed by fixation in 1% osmium tetroxide, additional washing with buffer, dehydration using ethanol, clearing with xylene, and air-drying. To conduct microscopic analysis, the samples were deposited on carbon tape, gold-coated, and examined using a scanning electron microscope (JEOL JSM-5400LV, Japan; Electron Microscope Unit, Assiut University, Assiut, Egypt) (Abd-Elrahman et al. 2024). This process aimed to detect and analyse helminths, including a detailed morphological examination of trematodes and cestodes, assessing the shape of the worms and their external structures such as spines, suckers, and genital pores (Halton 2004).

### Histopathological examination

For histopathological analysis, samples of liver and intestine were treated meticulously. They were fixed in 10% neutral buffered formalin, dehydrated in ascending concentrations of ethyl alcohol, and cleared in xylene for 24 hours. The samples were embedded in paraffin, sectioned at 4–5 μm, placed on glass slides, and stained with haematoxylin and eosin (Bancroft et al. 1994). The examination was performed using a light microscope (Olympus BX43F, Tokyo, Japan), and images were captured with a camera (Olympus EP50, Tokyo, Japan) at the Photomicrograph Lab of the Department of Parasitology, Faculty of Veterinary Medicine, Assiut University.

### Data analysis

Statistical analysis was performed using the epitools package in R software v4.2.2 (Team R 2010) to assess the risk factors for helminth infestation in cattle. Descriptive statistics compared the infested animal pattern based on seasonal differences (summer, autumn, winter, spring), age (<1 year, 1–3 years, >3 years), sex (female, male), and study site. Risk ratios (RR) and odds ratios (OR) with 95% confidence intervals (CI) were estimated to quantify the association between all the risk factors and the infestation, with summer, age < 1 year, female, and El-Dakhla as the reference categories. Statistical significance was determined by Wald chi-square tests (*P* < 0.05). A logistic regression model was used to determine the relationship between drug administration and different parasitic infestations. The model was constructed using the “GLM” function with a binomial distribution. The rate of infestation was computed by dividing the number of infected animals by the total number of animals tested and expressed as a percentage (Yousef et al. 2024).

## RESULTS

### Epidemiological analysis and risk factors of helminthic infestations in cattle

Of a total of 1 300 examined cattle, 784 (60.3%) animals were infested with one or more helminth species, with varying prevalence rates across different parasites. *Fasciola* spp. infestations were observed in 480 animals, accounting for 36.9% of the total infestations. *Moniezia* spp. had the highest infestation rate, affecting 610 (46.9%) cattle. *Paramphistoum* spp. was present in 348 (26.8%) cattle, while *Avitellina* spp. infestations accounted for 140 cases (10.8%) of the total infestations. The analysis of risk factors associated with helminthic infestations included variables such as season, age group, sex, and site, as displayed in Table 1.

**Table 1:** Epidemiological analysis and risk factors of helminthic infestations in cattle

Risk factor		Total	Non-infested	Infested (%)	Risk ratio	95% C.I (lower) 95% C.I. (upper)	Odds ratio	95% C.I. (lower) 95% C.I. (upper)	*P*-value
Season	summer	272	114	158 (58.1)	1	Ref*	1	Ref*	Ref*
	autumn	284	118	166 (58.5)	1.006 2	(0.874 1, 1.158 3)	1.015	(0.724, 1.423)	0.931
	winter	448	152	296 (66.1)	1.137 4	(1.008, 1.283 5)	1.404 5	(1.029 1, 1.916 3)	0.031 4
	spring	296	132	164 (55.4)	0.953 8	(0.826 2, 1.101 2)	0.896 7	(0.642 4, 1.250 7)	0.519 2

Age	<1	564	202	362 (64.2)	1	Ref*	1	Ref*	Ref*
	1 to 3	380	140	240 (63.2)	0.984	(0.891 7, 1.085 8)	0.956 5	(0.730 1, 1.254 6)	0.747 6
	>3	356	174	182 (51.1)	0.796 5	(0.707 3, 0.89 7)	0.584 1	(0.445 6, 0.764 9)	0.000 1

Sex	female	512	200	312 (60.9)	1	Ref*	1	Ref*	Ref*
	male	788	316	472 (59.9)	0.983	(0.898, 1.075)	0.958	(0.762, 1.202)	0.709

Site	El-Dakhla	240	164	76 (31.7)	1	Ref*	1	Ref*	Ref*
	Al-Farfra	220	20	200 (90.9)	2.871	(2.373, 3.473)	21.275	(12.717, 37.249)	<0.001
	El-Kharga	778	328	450 (57.8)	1.827	(1.503, 2.22)	2.955	(2.18, 4.034)	<0.001
	Baris	62	4	58 (93.5)	2.954	(2.426, 3.597)	29.906	(11.721, 103.535)	<0.001

Statistically significant *P*-value < 0.05

CI = confidence interval; Ref* = reference value

Seasonal variation revealed that winter had the highest infestation rate. Summer serves as the reference season with an infestation rate of 58.1%. Autumn is slightly higher at 58.5%, but the risk ratio (RR = 1.006 2) and odds ratio (OR = 1.015) both indicate no difference from summer. Winter shows a sharp increase in infestation rate to 66.1%, with its risk (RR = 1.137 4) and odds (OR = 1.404 5) being significantly higher. Spring, with a 55.4% infestation rate, has a risk ratio (RR = 0.953 8) and odds ratio (OR = 0.896 7) and shows a modestly lower risk compared to summer, but this is not statistically significant.

Infestation rates also vary significantly across age groups and regions, with less pronounced differences between sexes. Calves under one year of age have the highest infestation rate at 64.2%, serving as the reference group. Animals aged 1 to 3 years have a similar infestation rate at 63.2% (RR = 0.984, OR = 0.956 5), while those over 3 years show a significantly lower rate at 51.1% (RR = 0.796 5, OR = 0.584 1). Sex analysis reveals similar infestation rates between females (60.9%) and males (59.9%), with nearly identical risk and odds. Regionally, El-Dakhla has the lowest infestation rate at 31.7%, serving as the reference. Al-Farfra (90.9%, RR = 2.871, OR = 21.275) and Baris (93.5%, RR = 2.954, OR = 29.906) exhibit the highest rates and risks, with El-Kharga (57.8%, RR = 1.827, OR = 2.955) showing moderate infestation levels.

### Epidemiological distribution of different parasitic infestations

#### *FASCIOLA* SPECIES

The infestation rates of *Fasciola* spp. vary significantly with season, age, and sex. Infections are most prevalent during winter (45.09%), with a statistically significant increase compared to summer (28.68%), as indicated by an odds ratio (OR) of 2.038 7, *P* < 0.001. Autumn also shows a higher infestation rate (36.62%) with an OR of 1.435 6, *P* = 0.046. Age is another critical factor, with the youngest age group (<1 year) exhibiting the highest infestation rate (47.87%). This rate decreases significantly in older age groups: 1 to 3 years (32.63%, OR = 0.528, *P* < 0.001) and over 3 years (24.16%, OR = 0.347 5, *P* < 0.001). Sex does not significantly impact *Fasciola* spp. infestation rates, with females having an infestation rate of 38.28% compared to males at 36.04% (OR = 0.908 5, *P = *0.413, Table 2).

**Table 2:** The risk factors for *Fasciola* spp. infestations in cattle

Risk factor		Infested animals (%)	Odd ratio 95% CI (lower, upper)	*P*-value
Season	summer	78 (28.68)	1 (Ref*)	Ref*
	autumn	104 (36.62)	1.435 6 (1.005 3, 2.056)	0.046
	winter	202 (45.09)	2.038 7 (1.481, 2.824)	0.000 01
	spring	96 (32.43)	1.193 2 (0.834 1, 1.710 5)	0.332

Age	<1	270 (47.87)	1 (Ref*)	Ref*
	1 to 3	124 (32.63)	0.528 (0.402, 0.691 3)	3.21E-06
	>3	86 (24.16)	0.347 5 (0.258 1, 0.464 8)	6.33E-13

Sex	female	196 (38.28)	1 (Ref*)	Ref*
	male	284 (36.04)	0.91 (0.721 9, 1.144 1)	0.413 4

Statistically significant *P*-value < 0.05

CI = confidence interval; Ref* = reference value

#### *PARAMPHISTOMUM* SPECIES

*Paramphistomum* spp. infestation rates do not significantly differ across seasons, with rates ranging from 25.68% in winter to 33.33% in spring, and no season shows a statistically significant difference when compared to summer. Age, however, is a significant factor, with the highest infestation rate observed in the youngest group (<1 year) at 27.37%. The infection rate drops significantly in the 1- to 3-year age group to 15.73% (OR = 0.754, *P* = 0.052) and further in animals older than 3 years to 27.34% (OR = 0.374, *P* < 0.001). Sex does not show a significant impact on infestation rates, with females having a rate of 26.4% and males 44.85% (OR = 0.953, *P = *0.706) (Table 3).

**Table 3:** The risk factors for *Paramphistomum* spp. infestations in cattle

Risk factor		Infested animals (%)	Odd ratio 95% CI (lower, upper)	*P*-value
Season	summer	74 (27.21)	1 (Ref*)	Ref*
	autumn	78 (27.46)	1.013 1 (0.697, 1.473 3)	0.945
	winter	120 (26.79)	0.978 4 (0.697 8, 1.377 6)	0.902
	spring	76 (25.68)	0.924 4 (0.635 8, 1.344 3)	0.679

Age	<1	188 (33.33)	1 (Ref*)	Ref*
	1 to 3	104 (27.37)	0.754 (0.566, 1.002)	0.052
	>3	56 (15.73)	0.374 (0.266, 0.52)	<0.001

Sex	female	140 (27.34)	1 (Ref*)	Ref*
	male	208 (26.4)	0.953 (0.742, 1.226)	0.706

Statistically significant *P*-value < 0.05

CI = confidence interval; Ref* = reference value

#### *MONIEZIA* SPECIES

*Moniezia* spp. infection rates also showed no significant seasonal variation, with rates of 50% and 46.48% in winter and autumn, respectively, and slightly lower rates in spring (44.59%). Age does not significantly influence *Moniezia* spp. infestation rates, with rates of 48.94% in animals under 1 year, 44.74% in the 1- to 3-year group (OR = 0.845, *P* = 0.205), and 44.92% in animals over 3 years (OR = 0.891, *P* = 0.396). Sex impacts *Moniezia* spp. infestation slightly more, with females showing a higher infestation rate (50%) compared to males (44.92%), but this difference is not statistically significant (OR = 0.816, *P* = 0.074; Table 4).

**Table 4:** The risk factors for *Moniezia* spp*.* infestations in cattle

Risk factor		Infested animals (%)	Odd ratio 5% CI (lower, upper)	*P*-value
Season	summer	122 (44.85)	1 (Ref*)	Ref*
	autumn	132 (46.48)	1.068 (0.764, 1.492)	0.7
	winter	224 (50)	1.229 (0.908, 1.665)	0.18
	spring	132 (44.59)	0.99 (0.71, 1.379)	0.951

Age	<1	276 (48.94)	1 (Ref*)	Ref*
	1 to 3	170 (44.74)	0.845 (0.65, 1.097)	0.205
	>3	164 (46.07)	0.891 (0.683, 1.163)	0.396

Sex	female	256 (50)	1 (Ref*)	Ref*
	male	354 (44.92)	0.816 (0.653, 1.02)	0.074

Statistically significant *P*-value < 0.05

CI = confidence interval; Ref* = reference value

#### *AVITELLINA* SPECIES

*Avitellina* spp. infestation rates are lowest in spring (10.29%) and do not show significant seasonal variation, with slightly higher rates in winter (10.29%) and autumn (11.27%), but these differences are not statistically significant. Age is a significant factor, with the highest infestation rate in the 1- to 3-year group (13.16%, OR = 1.07, *P* = 0.734, decreasing significantly in animals under 1 year (12.41%) and further in animals over 3 years (5.62%, OR = 0.423, *P* = 0.007). Sex shows a significant difference, with females having a higher infestation rate (13.67%) compared to males (8.88%), with males showing a significantly lower rate (OR = 0.616, *P* = 0.007; Table 5).

**Table 5:** The risk factors for *Avitellina* spp. infestations in cattle

Risk factor		Infested animals (%)	Odd ratio 95% CI (lower, upper)	*P*-value
Season	summer	28 (10.29)	1 (Ref*)	Ref*
	autumn	32 (11.27)	1.106 (0.645, 1.905)	0.715
	winter	52 (11.61)	1.141 (0.706, 1.88)	0.594
	spring	28 (9.46)	0.911 (0.522, 1.589)	0.741

Age	<1	70 (12.41)	1 (Ref*)	Ref*
	1 to 3	50 (13.16)	1.07 (0.722, 1.576)	0.734
	>3	20 (5.62)	0.423 (0.246, 0.696)	0.007

Sex	female	70 (13.67)	1 (Ref*)	Ref*
	male	70 (8.88)	0.616 (0.433, 0.877)	0.007

Statistically significant *P*-value < 0.05

CI = confidence interval; Ref* = reference value

### Concurrent infestations between different parasites

**Figure 2:**
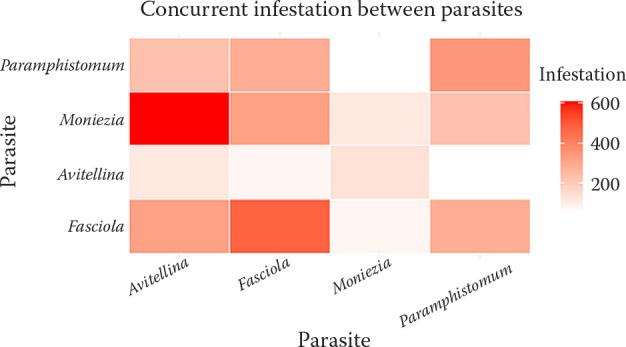
Displays the number of concurrent infestations between different pairs of parasites

The heatmap provides a clear visual representation of how often these parasites co-occur (Figure 2), *Fasciola* spp. is commonly co-infesting with other parasites, showing high counts of co-infestations with *Avitellina* spp. (82), *Moniezia* spp. (324), and *Paramphistomum* spp. (288). The highest co-infestation rate is between *Avitellina* spp. and *Moniezia* spp. (610), indicating a potential interaction or shared risk factor that promotes their simultaneous presence in hosts. Conversely, *Moniezia* spp. and *Paramphistomum* spp. have the lowest co-infestation rate (56).

### Association between deworming exposure and parasitic infestations in cattle

**Figure 3:**
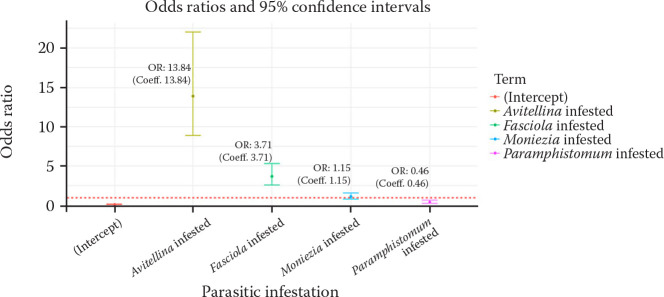
Odds ratios (OR) with 95% confidence intervals (CI), depicting the association between deworming exposure and parasitic infestations

The analysis of parasitic infestations reveals distinct differences in treatment response odds among cattle. *Fasciola* spp. infested cattle show significantly higher odds of responding to treatment, with an odds ratio of 3.711 (95% CI: 2.927 to 4.704, *P* < 0.001, *Paramphistomum* spp. infested cattle exhibit a substantially higher response likelihood, with an odds ratio of 13.829 (95% CI: 10.717 to 17.857, *P* < 0.001. In contrast, *Avitellina* spp. infestation is associated with significantly lower treatment response odds (OR: 0.456, 95% CI: 0.343 to 0.605, *P* < 0.001. *Moniezia* spp. infestation, however, does not significantly impact the treatment response, as indicated by an odds ratio of 1.146 (95% CI: 0.874 to 1.501, *P* = 0.403 (Table 6, Figure 3).

**Table 6:** Association between deworming exposure and parasitic infestations in cattle

Parasites	Non-infested	Infested	Rate of response (%)	95% CI (lower, upper)	*P*-value
Fasciola	126	170	57.4	(2.927, 4.704)	<0.001
Paramphistomum	206	90	30.4	(10.717, 17.857)	<0.001
Moniezia	110	186	56.8	(0.874, 1.501)	0.403
Avitellina	192	104	35.1	(0.343, 0.605)	<0.001

Statistically significant *P*-value < 0.05

CI = confidence interval

### Scanning electron microscopy of recovered helminths

**Figure 4:**
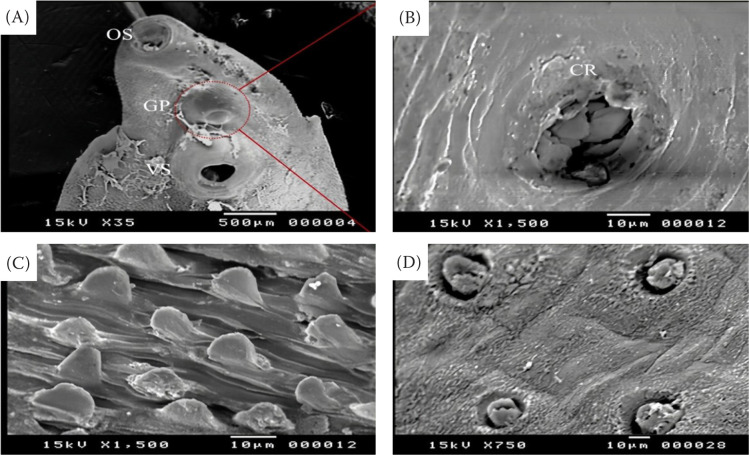
Surface morphology of adult *Fasciola* spp. showing anterior structures and tegumental architecture features Adult *Fasciola* species – (A) Ventral aspect of the anterior part, showing oral sucker (OS), ventral sucker (VS), and genital pore (GP; bar = 500 μm). (B) Genital pore (GP) with slightly protruded of cirrus (CR; bar = 10 μm). (C) The spines of the anterior region have finger-like projections at their tips (bar = 10 μm). (D) The tegument is covered with spines (bar = 10 μm)

Scanning electron microscopy of the recovered helminths provided valuable insights, as illustrated in Figure 4A. The surface topography of the adult *Fasciola* spp. revealed key features such as the oral sucker (OS), ventral sucker (VS), and the genital pore (GP). Figure 4B further highlights the genital pore (GP), observed with a slightly protruded cirrus (CR). This detailed examination significantly enhances our understanding of the anatomical characteristics of *Fasciola* spp. Notably, the anterior region of the parasite’s spines displays finger-like projections at their tips, resulting in a serrated appearance. Additionally, the surface of the parasite’s tegument between the spines is characterised by microridges and grooves (Figure 4C,D).

**Figure 5:**
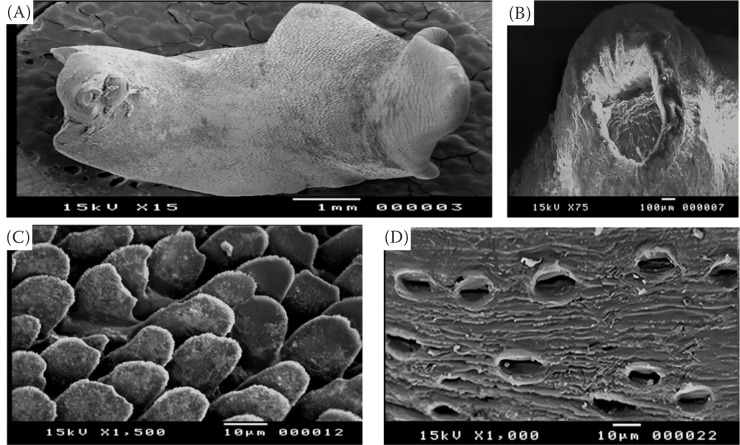
Surface structure changes in adult *Fasciola* spp. (A) Adult *Fasciola* species without visible changes (bar = 1 mm). (B) The oral sucker appears swollen with a smooth surface (bar = 100 μm). (C) Higher magnification reveals that some spines have lost finger-like projections (bar = 10 μm). (D) Dislodged spines expose their sockets in the syncytium (bar = 10 μm)

**Figure 6:**
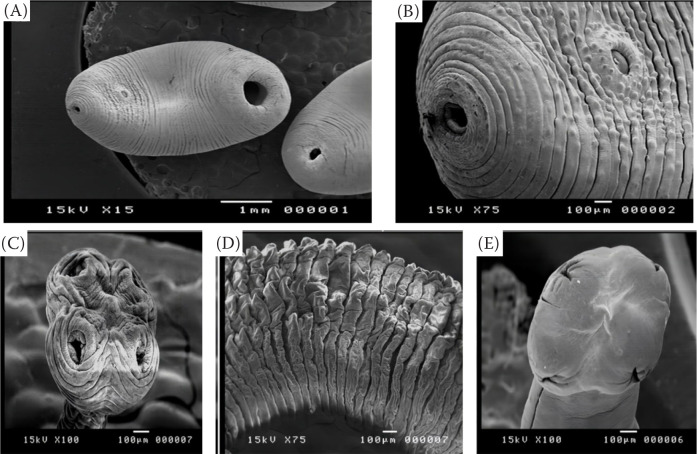
Morphological features of adult *Paramphistomum*, *Moniezia*, and *Avitellina* spp. (A,B) *Paramphistomum* species adult showing structures of oral sucker, ventral sucker, genital pore (GP) and tegumental folds (TF) (bar = 1 mm; 100 μm). (C) *Moniezia* sp. showing a globular scolex with four oval suckers. (D) *Moniezia* sp. proglottids (bar = 100 μm). (E) *Avitellina* species General anterior view of scolex, arrow show invaginated triangular suckers (bar = 100 μm)

For animals previously treated with anthelmintic drugs, slight surface alterations were observed in adult *Fasciola* spp. The adult *Fasciola* spp. do not exhibit any visible changes at low magnification. However, at higher magnification, slight alterations were observed, particularly in the oral sucker. The oral sucker appears swollen and has a smooth surface. Additionally, some spines have lost their finger-like projections, and dislodged spines expose their sockets in the syncytium, as illustrated in Figure 5. In contrast, the scanning did not reveal any alterations in the other helminth parasites, including *Paramphistomum* spp., *Moniezia* spp., and *Avitellina* spp., as shown in Figure 6.

### Pathological insights into parasitic infestation

#### GROSS EXAMINATION OF THE LIVER

**Figure 7:**
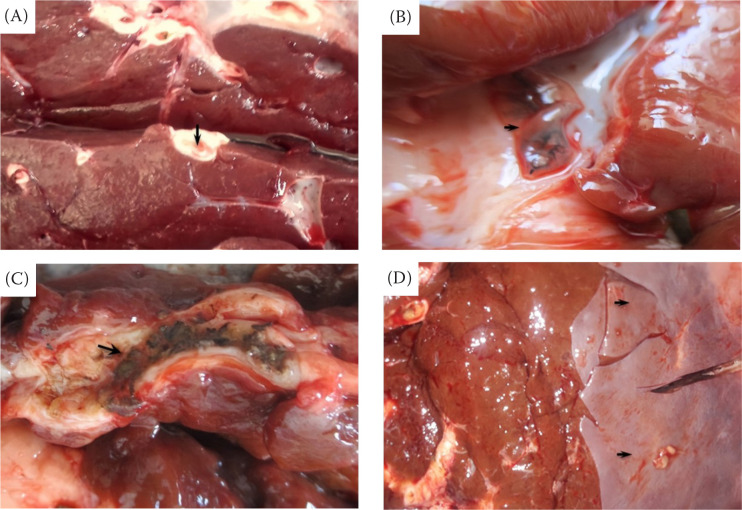
Illustration of the pathological impact of fascioliasis on the liver (A) *Fasciola* species within the bile duct, accompanied by extensive bleeding from the cut surface. (B) An infected liver exhibiting an adult *Fasciola* species adult worm. (C) The occurrence of haematoporphyrin pigment observed in the bile duct. (D) Illustration depicting engorgement of the bile duct

Upon pathological examination (see Figure 7), two distinct manifestations of fasciolosis, namely acute and chronic, were discerned. In cases of acute fasciolosis, macroscopic analysis of affected livers revealed notable features such as enlargement, firmness, congestion, and spontaneous bleeding from the incised surface. Mature liver flukes were frequently identified residing within the bile ducts (Figure 7A,B). Conversely, chronic fasciolosis was characterised by predominantly smaller liver sizes, firm consistency, and the presence of a corrugated capsule. Mature liver flukes were occasionally observed within the lumens of thickened bile ducts. Furthermore, the examination has shown the black minute granules, indicative of a haematoporphyrin pigment, with a palpable gritty sensation in the bile ducts. Additionally, certain normally sized livers exhibited regions of cirrhosis, accompanied by a thickened and calcified bile duct wall (Figure 7C,D).

#### GROSS EXAMINATION OF THE SMALL INTESTINE

**Figure 8:**
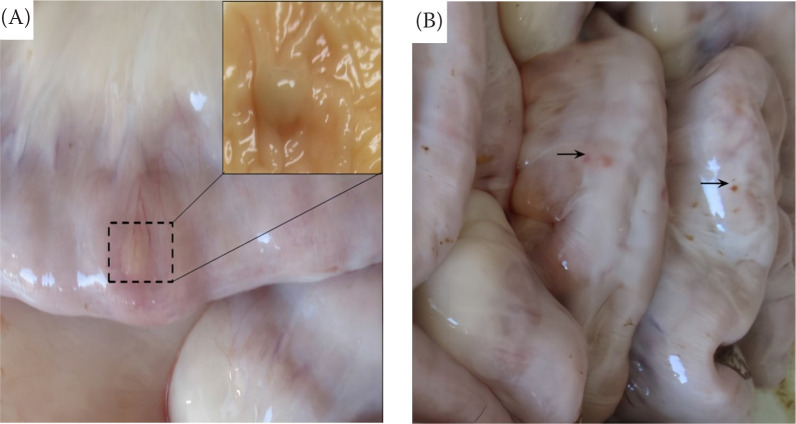
Illustration of the pathological consequences of* Moniezia* and *Avitellina* species infestations in the small intestine (A) Intestine showing nodules of various sizes. (B) Pinpointed haemorrhagic foci

The macroscopic evaluation of the intestine revealed several characteristics indicative of parasitic infestation, as shown in Figure 8.

The intestinal walls appeared thickened due to chronic inflammation and fibrosis, with visible nodules and raised areas on the mucosal surface. Some regions of the intestine were red and swollen, indicating inflammation. Additionally, there were erosions where the tapeworms had attached, causing localised damage to the intestinal lining, as illustrated in Figure 8.

### Histopathological examination

#### LIVER

**Figure 9:**
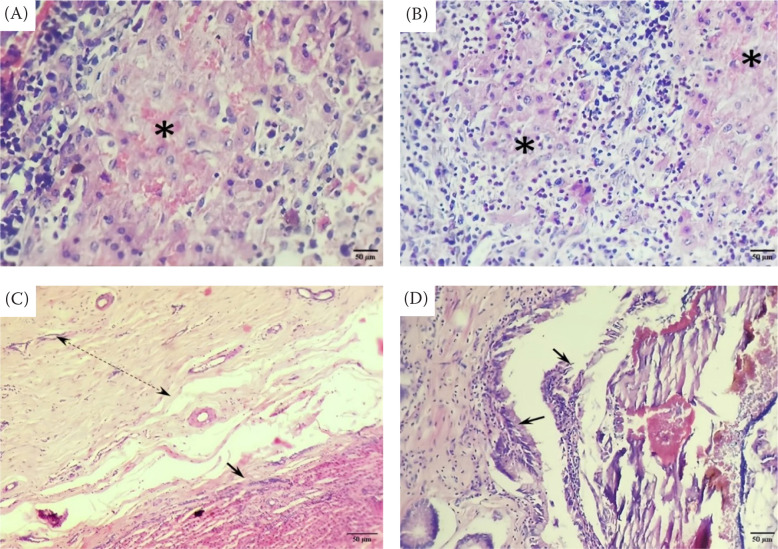
Histopathological examination of the liver tissue (haematoxylin and eosin staining) (A) Liver exhibiting the migratory tract of *Fasciola*, characterised by necrosis and haemorrhage (asterisk; bar = 50 μm). (B) Lymphocytic infiltration surrounding necrotic hepatic tissue (bar = 50 μm). (C) Chronic cholangitis and bile duct hyperplasia observed with epithelium desquamation double arrow, and inflammatory cell infiltration (arrow; bar = 50 μm). (D) Presence of dead and calcified parasite surrounded by fibrosis (arrow; bar = 50 μm)

In acute fasciolosis, a significant pathological manifestation involves the development of haemorrhagic migrating tracts resulting from the parasite’s travel through the liver. This intricate process includes the consumption of hepatocytes and erythrocytes, resulting in hepatocyte degeneration and subsequent destruction, as depicted in Figure 9A. The consequential interplay between hepatocyte degeneration and the presence of erythrocytes culminates in the creation of distinct haemorrhagic regions within the liver tissue, as visually represented in Figure 9B.

In contrast, chronic fasciolosis unveils unique characteristics within the liver. Notably, chronic cholangitis manifests itself as persistent irritation and inflammation of the bile ducts. This prolonged condition contributes significantly to the observed hyperplasia in the bile ducts with epithelium desquamation (Figure 9C). Adding to the complexity, the liver affected by chronic fasciolosis also highlights the presence of calcified deceased parasites, remnants of the fluke, which are surrounded by fibrosis (Figure 9D).

#### SMALL INTESTINE

**Figure 10:**
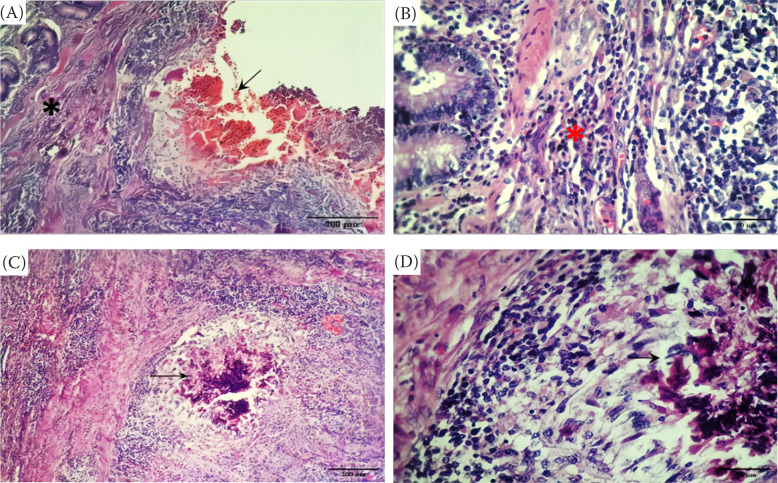
Histopathological analysis of intestinal changes (haematoxylin and eosin staining) (A,B) The presence of necrosis (arrow), and inflammatory cell reaction (asterisk; A, bar = 200 μm; B, bar = 50 μm). (C,D) A case with focal aggregation of lymphocytic reaction within the intestine, accompanied by fibrosis (arrow; C, bar = 200 μm; D, bar = 50 μm)

Histopathological examination of the small intestine, where both *Moniezia* spp. and *Avitellina* spp. were present, revealing significant pathological alterations induced by these parasites. These helminths cause necrosis, as visually depicted in Figure 10A,B. The image vividly portrays focal aggregation of lymphocytic reactions, highlighting the intricate immune response triggered by the parasitic activity. Additionally, the presence of inflammatory cell reactions is accompanied by fibrosis, indicating the onset of a chronic inflammatory process that persists over time, as shown in Figure 10C,D.

## DISCUSSION

The results present a comprehensive assessment of the prevalence of parasites in cattle, revealing significant variations due to seasonality, age, sex, and regional factors consistent with findings by Rizwan et al. (2023). Out of 1 300 cattle examined, 784 cases were detected to be infested, leading to a prevalence rate of 60.3%, higher than percentages reported in other Egyptian studies (20%) (Felefel et al. 2023). Winter recorded the highest infestation rate (66.1%), which was considerably higher than summer (58.1%), with notable risk ratios (RR = 1.137 4) and odds ratios (OR = 1.404 5). These strong seasonal trends align with findings that the transmission of the parasites is favoured during the wet season (Terfa et al. 2023). Age was a major determinant of infestation rates among cattle. Calves aged below 1 year had the highest infestation rate (64.2%), followed by cattle aged between 1 and 3 years (63.2%), with risk ratio (RR = 0.984) and odds ratios (OR = 0.956 5). Conversely, cattle over 3 years of age exhibited significantly lower infestation rates (51.1%) with respective risk and odds ratios (RR = 0.796 5, OR = 0.584 1), indicating lower susceptibility compared to the younger age groups, which concur with earlier observations (Kamel et al. 2024). Sex differences in infestation rates were minimal, with females having slightly higher (60.9%) rates than males (59.9%), but both sexes shared the same risk and odds ratios (RR = 0.983, OR = 0.958 for males), suggesting equal infestation risks. This finding aligns with Iranian studies, which also found no significant sex-based differences in infestation rates for cattle, *P* < 0.05 (Nouri et al. 2022). Regional analysis found significant differences with some regions, like Al-Farfra (90.9%) and Baris (93.5%) recording significantly higher infestation rates than others, like El-Dakhla (31.7%). These variations suggest potential associations with dominant climatic status and management conditions, which reflect the heterogeneous impact of parasitic infestations among the study cattle population (Parija 2022). The study of parasitic prevalence shows *Moniezia* spp. to be the most common parasite, with a prevalence rate of 46.9%.

Implicated in causing gastrointestinal disease in ruminants, *Moniezia* spp. poses a significant health risk to domestic animals, causing substantial financial loss (Kumar and Kaur 2023). Both geographical and species-specific variations in the prevalence of *Moniezia* spp. are high and vary from 3.69% in cattle to 94.10% in goats in Malaysia. *Fasciola* spp. emerged as the predominant trematode with 36.9 % prevalence, consistent with being the most common trematode of cattle in Nigeria (Ola-Fadunsin et al. 2020).

These findings emphasise the widespread and economic impact of *Fasciola* spp. infestations in ruminants, particularly *F. hepatica*, which consistently have high prevalence levels across different areas, necessitate effective control measures for animal health, thus minimising economic loss (Utrera-Quintana et al. 2022). *Paramphistomum* spp*.* had a higher prevalence of 26.8%, which was in contrast to its lower prevalence among trematodes in Ethiopia (9.5%) (Tiele et al. 2023). On the other hand, *Avitellina* spp. had the lowest prevalence at 10.8%. *Avitellina* spp., such as *Avitellina centripunctata* tapeworm, are worldwide-distributed intestinal parasites of ruminants and are most reported in Senegal (Ndom et al. 2019b). These variations show the differential prevalence rates and distributions of various parasitic species, thereby making the implementation of region-specific control measures in livestock production necessary.

The study is characterised by distinct patterns of parasitic infestations in cattle influenced by seasonality, age, and sex in *Fasciola* spp., *Paramphistomum* spp., *Moniezia* spp., and *Avitellina* spp*., Fasciola* spp. infestation takes place most commonly in autumn (36.62%) and remains relatively high in winter (45.09%), perhaps due to increased snail activity, the intermediate host, during the rainy seasons (Opio et al. 2021). Younger cattle, particularly those aged under 1 year, are more susceptible to *Fasciola* spp. (47.87%), while infestation decreases in the older age groups (1–3 years: 32.63%, >3 years: 24.16%). *Fasciola* spp. The infestation rate is not influenced by sex (Utrera-Quintana et al. 2022). *Paramphistomum* spp. infestations have a steady prevalence throughout seasons (winter: 25.68%, spring: 33.33%) and decrease in the 1–3 years age group (15.73%), as per previous findings supporting the high prevalence of *Paramphistomum* spp*.* amongst cattle in a high relative humidity, and the rainy season (Rafiq et al. 2022). *Moniezia* spp. infestations have equal frequencies by seasons (autumn: 44.48%, winter: 50%) with minor differences in age or sex, with findings showing peaks in October (autumn) and December (winter) (Sayed et al. 2024). *Avitellina* spp. infestations are somewhat independent of season (spring: 10.29%, autumn: 11.27%) but more frequent among 1–3-year-old cattle (13.16%) and slightly more frequent among females (13.67%) than among males (8.88%), like studies in goats showing no substantial difference in prevalence between rainy and dry seasons (Ndom et al. 2016a). The findings emphasise the importance of taking seasonal, age-group, and sex-related factors into account when designing effective measures to control parasitic infestations in cattle.

Co-occurrences have been reported in most previous studies, suggesting that these parasites are likely to co-occur (Spotts et al. 2020). This research has utilised the fact that *Fasciola* spp. and *Paramphistomum* spp. have high co-occurrence of infestations with each other and with *Moniezia* spp., the most prevalent concurrently occurring parasite, inferring they might share mutual hosts or environmental conditions that support co-infestation (Yacob et al. 2004). However, *Avitellina* spp. shows fewer co-occurring infestations with other parasites, suggesting variations in their epidemiology or with their hosts (Freeman et al. 2023). Overall, this heatmap provides a simplistic visual impression of how often these parasites co-occur. It can be useful for designing control measures that target specific combinations of them and for understanding their epidemiology.

The differentiation of treatment response probabilities by parasite indicates significant heterogeneity in deworming efficacy across helminth infestations in cattle. *Fasciola* spp. infested cows had much higher odds of being responsive to treatment (OR = 3.711, 95% CI: 2.927–4.704, *P* < 0.001, showing deworming interventions are highly effective against this parasite. This finding is similar to a Tanzanian study, where nitroxynil, oxyclozanide, closantel, and triclabendazole were effective against *Amphistome* infections and *Fasciola gigantica*, but albendazole was less effective (Nzalawahe et al. 2018).

In addition, the relatively extended prepatent period of flukes (10–12 weeks) would minimise the chance of identifying reinfection at slaughter, particularly when the treatment was done during the three months before culling (Kahl et al. 2021). Similarly, cattle infested with *Paramphistomum* spp. had exceedingly high treatment response odds (OR = 13.829, 95% CI: 10.717–17.857, *P* < 0.001), indicating their high susceptibility to the used anthelmintics. Contrary to findings in Bangladesh, where *Paramphistomum* spp. eggs were the most prevalent (25.5–56.4%) across different farming systems, this pattern suggests a persistent infection pressure and inadequate control interventions in some areas (Kouadio et al. 2021; Sayeed et al. 2024).

In contrast, *Avitellina* spp. infection was associated with significantly lower treatment response odds (OR = 0.456, 95% CI: 0.343–0.605, *P* < 0.001, indicating a reduced efficacy of commonly used treatments. This could be attributed to potential anthelmintic resistance, pharmacokinetic limitations, or intrinsic resilience of the parasite (Ndom et al. 2019a). Similarly, *Moniezia* spp. infections did not significantly impact the treatment response (OR = 1.146, 95% CI: 0.874–1.501, *P* = 0.403, suggesting comparable odds of treatment success relative to uninfested cattle. However, the treatment outcomes for *Avitellina* and *Moniezia* spp. must consider their relatively short prepatent periods (approximately six weeks); reinfestation occurring after treatment but before slaughter may result in detectable parasite presence at necropsy (Rehbein and Hamel 2025). These findings underscore the complex dynamics of post-treatment detection and highlight the growing concern over anthelmintic resistance (Salgado and Santos 2016; Ng’etich et al. 2023). Improper dosing, incorrect administration, and lack of monitoring can contribute to resistance development (Bosco et al. 2020). Hence, regular efficacy assessments, strategic drug rotation, and integrated parasite management plans are essential to ensure sustainable helminth control (Fissiha and Kinde 2021).

Numerous studies have employed advanced microscopy techniques to investigate the external morphologies of adult worms from various parasite species (Gomes et al. 2020). In line with this, our study employed scanning electron microscopy to examine the external morphology of adult helminths in both drug-exposed and non-exposed animals. We observed changes exclusively in *Fasciola* spp., particularly in the chronic stage.

In non-drug-exposed animals, typical characteristics were noted, such as the oral sucker, ventral sucker, and genital pore with slightly protruded cirrus. The anterior region of the parasite spines possesses finger-like processes at their tips, imparting a serrated character. The tegument surface of the parasite is also differentiated by microridges and grooves (Swarnakar and Damor 2016). The scanning electron microscopy (SEM) analysis of recovered helminths from previously dewormed animals, specifically focusing on adult *Fasciola* spp., yielded intriguing insights into their morphology and potential implications for parasite biology and host interactions (Gomes et al. 2020). At higher magnification, SEM images highlighted distinct alterations primarily localised to the oral sucker of adult *Fasciola* spp. align with a previous study in Egypt that investigated the activity of the commercially used flukicides against *Fasciola* spp. (Abdel-Fatah et al. 2022). These changes included a visibly swollen oral sucker with a smooth surface, indicative of potential physiological adaptations that could impact the parasite’s feeding behaviour and attachment mechanisms. The contrast between the smooth surface observed and its usual textured appearance suggests underlying structural modifications that may influence interactions with host tissues.

Moreover, SEM images revealed significant alterations in the spines of *Fasciola* spp., with some spines lacking their characteristic finger-like projections and exposing their sockets in the syncytium. These alterations were completely in similarity with the SEM of *Fasciola* spp. treated *in* *vitro* with different herbal extracts (Ullah et al. 2017). These observations are critical as spines are essential for anchoring *Fasciola* spp. to host tissues and maintaining stability during feeding (Fadavi et al. 2020). The structural damage observed, including the loss of projections and exposed sockets, may compromise the parasite’s ability to securely anchor within the host (Taibi et al. 2019).

In contrast, SEM examinations of other helminth parasites, such as *Paramphistomum* spp., *Moniezia* spp., and *Avitellina* spp., did not reveal morphological changes (Ndom et al. 2016b; Roy and Lyndem 2019). This stability in external morphology across these parasite species suggests that under similar conditions (Tihlarikova et al. 2016), their structural integrity remains relatively unchanged compared to the dynamic alterations observed in *Fasciola* spp. under SEM scrutiny (Ndom et al. 2016b; Roy and Lyndem 2019).

Pathological examination of the liver showed both acute and chronic fascioliasis. Acute fasciolosis was manifested as enlarged, congested livers with spontaneous haemorrhage. The presence of haemorrhagic migrating tracts was indicative of the hepatocyte and erythrocyte-destroying action of migrating parasites. Chronic fasciolosis, however, was characterised by smaller liver sizes with a corrugated capsule. Black granules and cirrhosis were present in some of the livers. Typically, chronic cholangitis and bile duct hyperplasia with calcified and dead parasites was observed (Sotohy et al. 2019; Ashoor and Wakid 2023).

In the intestine, the examination disclosed various-sized nodules with thickened patches on the mucosa. The presence of parasitic nodules induced necrosis and triggered lymphocytic reactions accompanied by fibrosis (Satish et al. 2018). This comprehensive investigation highlights the diverse pathological effects of helminth infections across multiple organs, offering detailed insights into their impact on the liver and intestines.

In conclusion, this study provides comprehensive insights into parasitic infestations affecting cattle in Upper Egypt. The prevalence of infestations varied significantly across seasons, with winter showing the highest rates. Age was identified as a crucial risk factor, with younger cattle more susceptible to certain parasites. Sex differences in infestation rates were minimal. Regional analysis highlighted stark contrasts in infestation prevalence, underscoring the need for localised management strategies. Pathological examinations revealed distinctive impacts on liver and intestinal health, with acute and chronic manifestations of fasciolosis observed. The severe liver and intestinal damage caused by *Fasciola* and frequent co-infestations with other parasites highlight the critical health risks posed by these infestations. Implementing more effective, parasite-specific treatments, particularly for *Fasciola* spp., and *Paramphistomum* spp., can significantly improve health and reduce the burden of infestation, while the low response rate for *Avitellina* calls for further research to enhance treatment efficacy. Additionally, due to the routine use of anthelmintics, future studies should assess potential drug resistance using appropriate diagnostic tools to guide sustainable parasite control. These results highlight the necessity of thorough deworming procedures along with targeted parasite control in reducing significant health hazards in cattle populations.

## RECOMMENDATIONS FROM THIS STUDY

In response to the issues identified, we propose several practical strategies for local farmers. These include the strategic rotation of anthelmintics and targeted treatment during high-risk seasons, particularly winter, to help prevent drug resistance. Improving pasture hygiene and waste management can reduce environmental contamination. The education of farmers on biosecurity and integrated parasite control will support more effective long-term management.

Additionally, we recommend seasonal snail control and habitat modification in areas susceptible to fluke transmission. Finally, establishing sustainable parasite monitoring programs, including resistance surveillance, is essential for maintaining treatment efficacy and enabling early detection of emerging resistance. These combined approaches provide a sustained means of lowering parasitic infestation and enhancing the health of cattle when customised to local conditions.
